# Is Lymph Node Dissection Necessary During Radical Nephroureterectomy for Clinically Node-Negative Upper Tract Urothelial Carcinoma? A Multi-Institutional Study

**DOI:** 10.3389/fonc.2022.791620

**Published:** 2022-04-29

**Authors:** Hsiang-Ying Lee, Chao-Hsiang Chang, Chi-Ping Huang, Chih-Chin Yu, Chi-Wen Lo, Shiu-Dong Chung, Wei-Che Wu, I-Hsuan Alan Chen, Jen-Tai Lin, Yuan-Hong Jiang, Yu-Khun Lee, Thomas Y. Hsueh, Allen W. Chiu, Yung-Tai Chen, Chang-Min Lin, Yao-Chou Tsai, Wei-Chieh Chen, Bing-Juin Chiang, Hsu-Che Huang, Chung-Hsin Chen, Chao-Yuan Huang, Chia-Chang Wu, Wei Yu Lin, Jen-Shu Tseng, Hung-Lung Ke, Hsin-Chih Yeh

**Affiliations:** ^1^Department of Urology, Kaohsiung Medical University Hospital, Kaohsiung, Taiwan; ^2^Department of Urology, School of Medicine, College of Medicine, Kaohsiung Medical University, Kaohsiung, Taiwan; ^3^Department of Urology, Kaohsiung Municipal Ta-Tung Hospital, Kaohsiung, Taiwan; ^4^Graduate Institute of Medicine, College of Medicine, Kaohsiung Medical University, Kaohsiung, Taiwan; ^5^Department of Urology, China Medical University and Hospital, Taichung, Taiwan; ^6^School of Medicine, China Medical University, Taichung, Taiwan; ^7^Division of Urology, Department of Surgery, Taipei Tzu Chi Hospital, The Buddhist Medical Foundation, New Taipei City, Taiwan; ^8^School of Medicine, Buddhist Tzu Chi University, Hualien, Taiwan; ^9^Graduate Program in Biomedical Informatics, College of Informatics, Yuan-Ze University, Chung-Li, Taiwan; ^10^Division of Urology, Department of Surgery, Far Eastern Memorial Hospital, New Taipei City, Taiwan; ^11^Institute of Biomedical Engineering, National Taiwan University, Taipei, Taiwan; ^12^Division of Urology, Department of Surgery, Kaohsiung Veterans General Hospital, Kaohsiung, Taiwan; ^13^Department of Urology, Hualien Tzu Chi Hospital, Buddhist Tzu Chi Medical Foundation and Tzu Chi University, Hualien, Taiwan; ^14^Division of Urology, Department of Surgery, Taipei City Hospital Renai Branch, Taipei, Taiwan; ^15^Department of Urology, School of Medicine, National Yang Ming Chiao Tung University, Taipei, Taiwan; ^16^College of Medicine, National Yang Ming Chiao Tung University, Taipei, Taiwan; ^17^Department of Urology, Taiwan Adventist Hospital, Taipei, Taiwan; ^18^Department of Urology, School of Medicine, College of Medicine, Taipei Medical University, Taipei, Taiwan; ^19^Department of Urology, Taipei Medical University Hospital, Taipei, Taiwan; ^20^College of Medicine, Fu-Jen Catholic University, New Taipei City, Taiwan; ^21^Department of Urology, Cardinal Tien Hospital, New Taipei City, Taiwan; ^22^Department of Life Science, College of Science, National Taiwan Normal University, Taipei, Taiwan; ^23^Department of Urology, National Taiwan University Hospital, College of Medicine, National Taiwan University, Taipei, Taiwan; ^24^Department of Urology, Shuang Ho Hospital, Taipei Medical University, New Taipei City, Taiwan; ^25^TMU Research Center of Urology and Kidney (TMU-RCUK), Taipei Medical University, Taipei, Taiwan; ^26^Division of Urology, Department of Surgery, Chang Gung Memorial Hospital, Chiayi, Taiwan; ^27^Department of Medicine, College of Medicine, Chang Gung University, Taoyuan, Taiwan; ^28^Department of Medicine, Chang Gung University, Taoyuan, Taiwan; ^29^Department of Urology, MacKay Memorial Hospital, Taipei, Taiwan; ^30^Department of Urology, Mackay Medical College, New Taipei City, Taiwan; ^31^Institute of Biomedical Informatics, National Yang Ming Chiao Tung University, Taipei, Taiwan

**Keywords:** lymph node dissection, clinical lymph node negative, muscle-invasive stage, pathological lymph node positive, upper tract urothelial carcinoma

## Abstract

**Purpose:**

This study aimed to compare the oncological outcomes of patients with upper tract urothelial carcinoma (UTUC) without clinical lymph node metastasis (cN0) undergoing lymph node dissection (LND) during radical nephroureterectomy (NU).

**Methods:**

From the updated data of the Taiwan UTUC Collaboration Group, a total of 2726 UTUC patients were identified. We only include patients with ≥ pT2 stage and enrolled 658 patients. The Kaplan–Meier estimator and Cox proportional hazards model were used to analyze overall survival (OS), cancer-specific survival (CSS), disease-free survival (DFS), and bladder recurrence-free survival (BRFS) in LND (+) and LND (−) groups.

**Results:**

A total of 658 patients were included and 463 patients without receiving LND and 195 patients receiving LND. From both univariate and multivariate survival analysis, there are no significant difference between LND (+) and LND (-) group in survival rate. In LND (+) group, 18.5% patients have pathological LN metastasis. After analyzing pN+ subgroup, it revealed worse CSS (p = 0.010) and DFS (p < 0.001) compared with pN0 patients.

**Conclusions:**

We found no significant survival benefit related to LND in cN0 stage, ≥ pT2 stage UTUC, irrespective of the number of LNs removed, although pN+ affected cancer prognosis. However, from the result of pN (+) subgroup of LND (+) cohort analysis, it may be reasonable to not perform LND in patients with cT2N0 stage due to low positive predictive value of pN (+). In addition, performing LND may be considered for ureter cancer, which tends to cause lymphatic and hematogenous tumor spreading. Further large prospective studies are needed to validate our findings.

## Introduction

Upper tract urothelial carcinoma (UTUC), comprising renal pelvis and ureter cancer, has a higher incidence and female predominance in Taiwan than in other countries. In Taiwan, UTUC accounts for 40% of urothelial carcinomas (UCs), while it accounts for approximately 5–10% of UCs in Western countries (1–3). Radical nephroureterectomy (NU) with bladder cuff excision is the standard treatment for non-metastatic UTUC (3). According to previous studies, approximately 30–40% of lymph node involvement is discovered at the time of surgery (4); however, the percentage of patients receiving lymph node dissection (LND) varies widely. In a large population cohort of 16,619 UTUCs, 15.4% of patients underwent LND (5). Chappidi et al. revealed that the trend of LND increased from 20% (60/295) in 2004 to 33% (106/320) in 2012, which may reflect that LND is gradually becoming more acceptable for surgeons in clinical practice (6). In our recently published study, we also discovered different proportions of LND in different minimally invasive NU approaches, and robot-assisted NU had the highest LND rate (41.1%). The surgical technique and experience of surgeons also affects the rate of LND (7).

Although the benefits of LND are well-established in muscle-invasive bladder cancer (BC), the role of routine concomitant LND in UTUC, which is considered to have a similar histology and phenotype, is still controversial (8). Some physicians hypothesized that performing LND provides more accurate pathological disease staging and potentially better oncological outcomes, especially at higher T stages of UTUC (9, 10). Pathologically, lymph node metastasis is a poor prognostic factor for survival in UTUC, which might strengthen the rationale for performing LND in UTUC (11). However, it is not known if LND is clinically beneficial for patients without lymph node involvement. Nevertheless, LND may result in a higher risk of postoperative complications. A previous study demonstrated that patients receiving LND have a higher rate of hemorrhagic complications because the lymph nodes are near the great vessels (5, 12).

In the present study, based on a large retrospective cohort from multiple institutions in Taiwan, we aimed to resolve the issue of whether LND is necessary for UTUC patients with clinical node-negative status (cN0) on imaging studies before radical surgery.

## Materials and Methods

### Patient Collection

This study was approved by our Institutional Review Board (KMUHIRB-E(I)-20180214). We retrospectively reviewed the updated data from 15 participating hospitals under the Taiwan UTUC Collaboration Group and identified 2767 patients with UTUC. We excluded patients who did not receive NU (n = 480) and those with pathological T1 (pT1) or pTis stage disease (n = 1088). Patients with cN (+) (n = 328) disease or those lacking any variables of interest or who were lost to follow-up (n = 213) were also excluded. Finally, we included 658 patients with clinical N0 status who received NU between July 2001 and February 2021. Patients were divided into groups with and without LND (defined as LND (+) or LND (−), respectively).

In addition to the LND (+) and (−) groups, various variables were collected for analysis, including age, gender, history of BC, preoperative hydronephrosis, tumor location, tumor size, tumor focality, and important pathological features such as tumor grade, pT stage, histological variant, and lymphovascular invasion (LVI).

### Definitions and Endpoints

Pathological tumor staging was based on specimens obtained after NU, with or without LND, according to the 2010 TNM (tumor, node, and metastasis) classification, and the tumor grade was defined according to the 2004 World Health Organization/International Society of Urologic Pathology consensus classification. Regular follow-up strategies follow standard guidelines. The endpoint was to compare the survival outcomes including overall survival (OS), cancer-specific survival (CSS), disease-free survival (DFS), and bladder recurrence-free survival (BRFS) between the LND (+) and LND (−) groups. The cause of death was determined by the attending doctor or death certificate.

### Statistical Analysis

To compare differences between groups, we used Student’s t-test for continuous variables and Pearson’s chi-squared test for categorical variables. The Kaplan–Meier estimator was used to estimate the rates of prognostic outcomes, and survival curves were compared using the stratified log-rank test. The Cox proportional hazards model was selected to evaluate the impact of LND on prognosis, with or without correction for confounding factors. IBM SPSS Statistics software version 26 was used for the analysis. All statistical analyses were two-tailed, and p < 0.05, was considered significant.

## Results

We compared the basic clinical and pathological characteristics of patients undergoing RNU between LND (+) and LND (−) groups (**Table 1**). A total of 658 patients were included in this study. Overall, 463 patients did not receive LND, and 195 patients received LND. The median number of LN removed is 4. There were significant differences in age (p = 0.022), Eastern Cooperative Oncology Group (ECOG) scores (p < 0.001), histological variants (p = 0.008), and pT stage (p < 0.001).

**Table 1:** Clinicopathological data of cN0 UTUC patients receiving nephroureterectomy.

Variables	LND (-) (N=463)		LND (+) (N=195)		*p* value^a^
	N	%	N	%	
Gender					0.166
Men	196	(42.3)	94	(48.2)	
Women	267	(57.7)	101	(51.8)	
Age^b^ Mean ±SD	69.8±10.8		67.7±10.6		0.022*
ECOG scores					<0.001**
0	188	(40.6)	40	(20.5)	
1	214	(46.2)	130	(66.7)	
2	47	(10.2)	23	(11.8)	
3	9	(1.9)	2	(1.0)	
4	5	(1.1)	0	(0.0)	
Tumor location					0.466
Renal pelvis	217	(47.1)	94	(48.5)	
Ureter	163	(35.4)	60	(30.9)	
Synchronous	81	(17.6)	40	(20.6)	
Tumor size					0.266
non-visible	2	(0.4)	1	(0.5)	
<1cm	14	(3.0)	3	(1.5)	
≥1 & < 2 cm	81	(17.5)	33	(16.9)	
≥2 & < 3 cm	117	(25.3)	37	(19.0)	
≥ 3cm	249	(53.8)	121	(62.1)	
Histological variant					0.008**
No	409	(88.3)	157	(80.5)	
Yes	54	(11.7)	38	(19.5)	
Tumor grade					0.166
Low grade	35	(7.6)	9	(4.6)	
High grade	427	(92.4)	186	(95.4)	
Multiplicity					0.184
No	298	(64.3)	113	(58.5)	
Yes	164	(35.7)	80	(41.5)	
Lymphovascular invasion					0.474
No	315	(69.7)	129	(66.8)	
Yes	137	(30.3)	64	(33.2)	
Preoperative hydronephrosis					0.089
No	150	(32.8)	77	(39.7)	
Yes	308	(67.2)	117	(60.3)	
History of BC		–		–	0.379
No	382	(82.5)	158	(81.0)	
Previous BC	22	(4.8)	6	(3.1)	
Concurrent BC	59	(12.7)	31	(15.9)	
Pathological stage T					<0.001**
pT2	195	(42.3)	47	(24.2)	
pT3	248	(53.8)	99	(51.0)	
pT4	18	(3.9)	48	(24.7)	
Clavien-Dindo classification					0.683
No	275	(60.0)	118	(61.8)	
Grade I	61	(13.3)	29	(15.2)	
Grade II	85	(18.6)	34	(17.8)	
Grade III	18	(3.9)	4	(2.1)	
Grade IV	8	(1.7)	4	(2.1)	
Grade V	11	(2.4)	2	(1.0)	
Post-OP Complication					
ESRD	62	(13.9)	19	(9.9)	0.170
Ileus	13	(2.9)	4	(2.1)	0.556
Follow up (months)^c^ median	33.5		24.2		0.049*

a Chi-Squared test calculated for the difference Variables.

b Student’s t-test calculated for the difference in means. * < 0.05, ** < 0.01.

c Wilcoxon rank-sum test calculated for the difference in medians. * < 0.05, ** < 0.01.

UTUC, upper tract urothelial carcinoma; LND, lymph node dissection; BC, bladder cancer; ESRD, end-stage renal disease; NU, nephroureterectomy.

### Survival Outcomes (OS, CSS, DFS)

**Figure 1:**
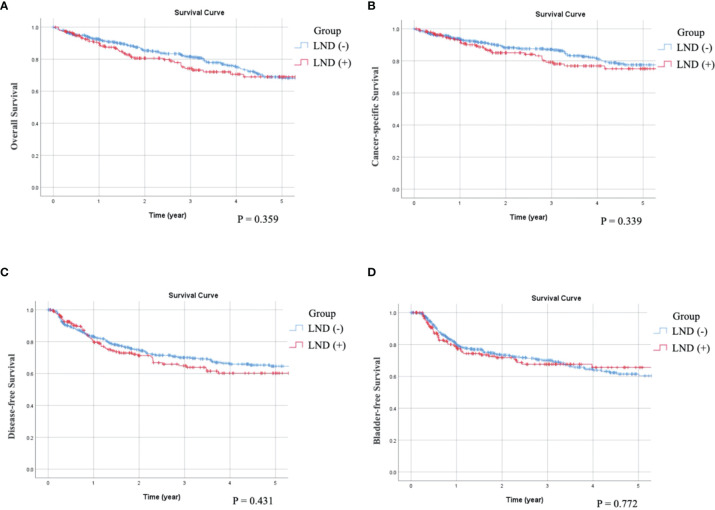
Compare Kaplan-Meier curves between patients without receiving LND (LND (-)) or with receiving LND (LND (+)) by log-rank test. **(A)** Overall survival, p = 0.359. **(B)** Cancer-specific survival, p = 0.339. **(C)** Disease-free Survival, p = 0.431. **(D)** Bladder recurrence-free survival, p = 0.772.

**Figure 2:**
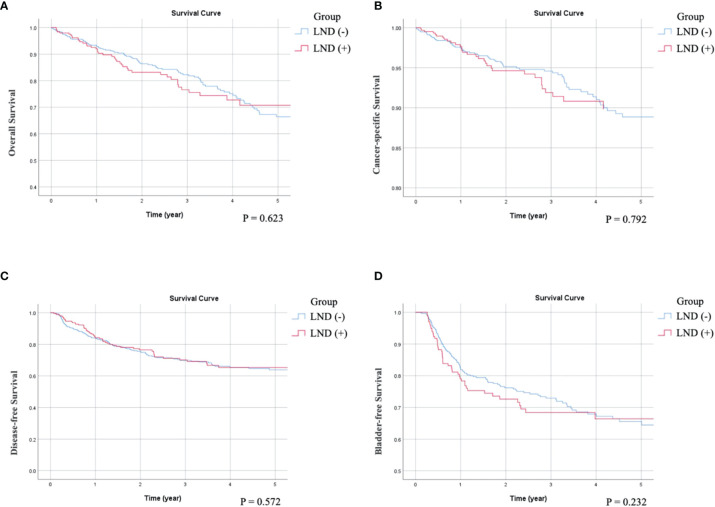
Compare Kaplan-Meier curves between patients without receiving LND (LND (-)) or with receiving LND (LND (+)) after adjusting variables. **(A)** Overall survival, p = 0.623. **(B)** Cancer-specific survival, p = 0.792. **(C)** Disease-free Survival, p = 0.572. **(D)** Bladder recurrence-free survival, p = 0.232.

As can be seen in the univariate survival analysis shown in **Table 2**, LND status was not associated with OS, CSS, or DFS. The overall 5-year OS rate in LND (−) patients was 68% and that of LND (+) patients was 69%. The 5-year CSS rates were 77% for LND (−) and 75% for LND (+) patients. The 5-year DFS rates were 64% for LND (−) and 60% for LND (+) patients. Kaplan–Meier analysis showed that OS (p = 0.359), CSS (p = 0.339) and DFS (p = 0.431) were not significant difference between LND (–) and LND (+) (**Figures 1A-C****)**. Furthermore, multivariate survival analysis and adjusted 5-year survival rates indicated that LND (+) was not associated with better survival outcomes (OS, p = 0.672; CSS, p = 0.770; and DFS, p = 0.489) (**Table 3**). Kaplan–Meier analysis also showed insignificant impact on survival (OS: p = 0.623; CSS: p = 0.792; DFS: p = 0.572) (**Figures 2A-C**). Regarding OS, age (p = 0.003, p = 0.016), ECOG status (p < 0.001, p = 0.001), previous BC (p = 0.008, p = 0.006), preoperative hydronephrosis (p < 0.001, p < 0.001), and pT4 stage (p < 0.001, p < 0.001) were significant in both univariate and multivariate analyses, respectively. Regarding CSS, ECOG (p < 0.001, p = 0.047), previous BC (p = 0.018, p = 0.008), concurrent BC (p = 0.002, p = 0.020), preoperative hydronephrosis (p = 0.005, p < 0.001), and pT4 stage (p < 0.001, p < 0.001) were significantly different in both univariate and multivariate analyses, respectively. Regarding DFS, concurrent BC (p < 0.001, p = 0.001), LVI (p < 0.001, p < 0.001), tumor grade (p = 0.007, p = 0.023), and pT4 stage (p < 0.001, p < 0.001) were significantly different in both univariate and multivariate survival analyses, respectively.

**Table 2:** Comparative univariate survival analysis of UTUC patients receiving NU.

Univariate analysis		OS				CSS				DFS				BRFS			
		HR (95% CI)		p-value		HR (95% CI)		p-value		HR (95% CI)		p-value		HR (95% CI)		p-value	
Group				0.396				0.340				0.432				0.772	
LND (-)		1				1				1				1			
LND (+)		1.165 (0.818, 1.659)				1.222 (0.810, 1.844)				1.131 (0.833, 1.535)				1.049 (0.758, 1.451)			
Sex				0.901				0.694				0.395				<0.001**	
Male		1				1				1				1			
Female		0.980 (0.710, 1.352)				0.927 (0.634, 1.354)				0.885 (0.669, 1.172)				0.573 (0.426, 0.770)			
Age				0.003**				0.172				0.419				0.770	
<70		1				1				1				1			
>=70		1.633 (1.178, 2.263)				1.304 (0.891, 1.907)				1.123 (0.848, 1.488)				1.045 (0.779, 1.400)			
Histological variant				0.043*				0.120				0.070				0.729	
No		1				1				1				1			
Yes		1.540 (1.014, 2.337)				1.482 (0.902, 2.434)				1.420 (0.972, 2.075)				0.924 (0.592, 1.444)			
ECOG scores				<0.001**				<0.001**				0.014*				0.836	
0~1		1				1				1				1			
2~4		2.905 (1.991, 4.238)				2.465 (1.549, 3.923)				1.634 (1.105, 2.415)				0.951 (0.591, 1.531)			
Tumor size																	
<1cm		1				1				1				1			
≥1 & < 2 cm		2.742 (0.649, 11.576)		0.170		3.485 (0.463, 26.248)		0.225		1.082 (0.373, 3.142)		0.885		0.850 (0.377, 1.918)		0.696	
≥2 & < 3 cm		2.199 (0.525, 9.209)		0.281		3.180 (0.428, 23.618)		0.258		1.481 (0.531, 4.131)		0.453		0.776 (0.349, 1.728)		0.535	
≥ 3cm		2.935 (0.722, 11.932)		0.132		4.166 (0.577, 30.055)		0.157		1.987 (0.734, 5.380)		0.177		0.769 (0.357, 1.657)		0.502	
Tumor location																	
Renal pelvis		1				1				1				1			
Ureter		1.361 (0.947, 1.957)		0.096		1.307 (0.849, 2.012)		0.223		1.217 (0.885, 1.647)		0.227		1.275 (0.911, 1.785)		0.156	
Synchronous		1.442 (0.940, 2.213)		0.094		1.489 (0.905, 2.449)		0.117		1.397 (0.964, 2.023)		0.077		1.829 (1.255, 2.664)		0.002**	
Multiplicity				0.026*				0.004**				0.006**				0.001**	
No		1				1				1				1			
Yes		1.447 (1.045, 2.003)				1.761 (1.202, 2.578)				1.494 (1.124, 1.987)				1.671 (1.241, 2.249)			
History of BC																	
No		1				1				1				1			
Previous BC		2.332 (1.253, 4.338)		0.008**		2.422 (1.167, 5.024)		0.018*		1.640 (0.888, 3.028)		0.114		3.214 (1.881, 5.493)		<0.001**	
Concurrent BC		1.749 (1.140, 2.682)		0.010*		2.117 (1.317, 3.405)		0.002**		1.999 (1.398, 2.860)		<0.001**		1.926 (1.314, 2.823)		0.001**	
Preoperative hydronephrosis				<0.001**				0.005**				0.088				0.383
No		1				1				1				1		
Yes		2.109 (1.405, 3.167)				1.966 (1.229, 3.147)				1.311 (0.960, 1.790)				1.151 (0.839, 1.577)		
Lymphovascular invasion				0.019*				0.002**				<0.001**				0.112
No		1				1				1				1		
Yes		1.502 (1.071, 2.107)				1.839 (1.242, 2.722)				1.979 (1.485, 2.639)				0.758 (0.539, 1.067)		
Tumor grade				0.035*				0.044*				0.007**				0.013*
Low grade		1				1				1				1		
High grade		2.609 (1.069, 6.364)				22.925 (1.087, 483.560)				3.405 (1.401, 8.276)				0.566 (0.363, 0.885)		
Pathological stage T																
pT2		1				1				1				1		
pT3		1.645 (1.150, 2.352)		0.006**		2.098 (1.341, 3.281)		0.001**		1.899 (1.376, 2.623)		<0.001**		0.889 (0.661, 1.194)		0.433
pT4		3.429 (1.881, 6.251)		<0.001**		4.891 (2.471, 9.681)		<0.001**		3.906 (2.297, 6.641)		<0.001**		0.206 (0.051, 0.836)		0.027*

Cl, confidence; HR, hazard ratio; OS, overall survival; CSS, cancer-specific survival; DFS, disease-free survival; BRFS, Bladder Recurrence-free survival.

* < 0.05, ** < 0.01.

UTUC, upper tract urothelial carcinoma; LND, lymph node dissection; BC, bladder cancer; ESRD, end-stage renal disease; NU, nephroureterectomy.

**Table 3:** Comparative multivariate survival analysis of UTUC patients receiving NU.

Multivariable analysis	OS		CSS		DFS		BRFS	
	HR (95% CI)	p-value	HR (95% CI)	p-value	HR (95% CI)	p-value	HR (95% CI)	p-value
Group		0.672		0.770		0.489		0.170
LND (-)	1		1		1		1	
LND (+)	1.086 (0.742, 1.588)		1.069 (0.684, 1.671)		0.889 (0.637, 1.241)		1.272 (0.902, 1.793)	
Age		0.016*		0.309		0.483		0.446
<70	1		1		1		1	
>=70	1.552 (1.084, 2.221)		1.244 (0.817, 1.894)		1.116 (0.822, 1.515)		1.129 (0.827, 1.541)	
Histological variant		0.434		0.792		0.307		0.855
No	1		1		1		1	
Yes	1.201 (0.759, 1.899)		1.077 (0.621, 1.866)		1.238 (0.822, 1.863)		0.955 (0.582, 1.568)	
ECOG scores		0.001**		0.047*		0.237		0.642
0~1	1		1		1		1	
2~4	2.061 (1.363, 3.117)		1.676 (1.008, 2.788)		1.287 (0.847, 1.957)		0.886 (0.532, 1.476)	
Multiplicity		0.256		0.202		0.239		0.030*
No	1		1		1		1	
Yes	1.232 (0.859, 1.767)		1.318 (0.863, 2.014)		1.208 (0.882, 1.655)		1.446 (1.035, 2.020)	
History of BC								
No	1		1		1		1	
Previous BC	2.502 (1.294, 4.838)	0.006*	2.887 (1.323, 6.301)	0.008*	1.650 (0.869, 3.133)	0.126	2.829 (1.602, 4.997)	<0.001**
Concurrent BC	1.523 (0.953, 2.434)	0.079	1.865 (1.105, 3.147)	0.020*	1.952 (1.317, 2.892)	0.001**	1.581 (1.022, 2.446)	0.040*
Preoperative hydronephrosis		< 0.001**		< 0.001**		0.011*		0.457
No	1		1		1		1	
Yes	2.617 (1.697, 4.035)		2.522 (1.518, 4.191)		1.526 (1.100, 2.116)		1.132 (0.816, 1.571)	
Lymphovascular invasion		0.136		0.035		< 0.001**		0.449
No	1		1		1		1	
Yes	1.317 (0.917, 1.891)		1.565 (1.032, 2.374)		1.744 (1.289, 2.361)		0.872 (0.611, 1.244)	
Tumor grade		0.161				0.023*		0.026*
Low grade	1				1		1	
High grade	1.921 (0.771, 4.784)				2.846 (1.158, 6.995)		0.589 (0.369, 0.938)	
pathological stage T								
pT2	1		1		1		1	
pT3	1.530 (1.043, 2.244)	0.030*	1.859 (1.151, 3.002)	0.011*	1.737 (1.236, 2.440)	0.001**	0.875 (0.636, 1.203)	0.410
pT4	3.939 (1.985, 7.817)	<0.001**	5.038 (2.307, 11.000)	<0.001**	3.252 (1.770, 5.975)	<0.001**	0.127 (0.017, 0.928)	0.042*

Cl, confidence; HR, hazard ratio; OS, overall survival; CSS, cancer-specific survival; DFS, disease-free survival; BRFS, Bladder Recurrence-free survival.

* < 0.05, ** < 0.01.

### Bladder Recurrence (BRFS)

There was no statistically significant difference between the LND (−) and LND (+) groups in terms of BRFS in both univariate and multivariate analyses. The overall 5-year BRFS rate was 62% in LND (−) and 66% in LND (+) patients (**Tables 2**, **3**, and **Figures 1D**, **2D**). Additionally, multiplicity (p = 0.001 and 0.030, respectively), previous BC (p < 0.001 and p < 0.001, respectively), concurrent BC (p = 0.001 and p = 0.040, respectively), and pT4 stage (p = 0.027 and p = 0.042, respectively) significantly correlated with BRFS in both analyses.

## Discussion

Whether routine LND at the time of NU should be performed in non-metastatic UTUC patients has always been debated, especially for patients with cN0 status. Previous studies have demonstrated the benefits of LND for oncological outcomes in advanced UTUC. Furuse et al. found that removal of defined systematic regional nodal areas can improve survival in cTanyN0M0, but that there was no significant benefit at the pTis-1 stage (13). Similar results were also observed in Dong’s research; LND was associated with a better survival benefit in cN0 patients, especially in the muscle invasive stage. Even after receiving adjuvant therapy, patients receiving LND still have better outcomes than those who do not receive LND (14). In contrast, Inokuchi et al. indicated that there was no therapeutic benefit of LND, even in clinically advanced T stage disease (15). According to the latest EAU 2020 guidelines, LND is unnecessary in cases of non-muscle invasive disease due to the low risk of LN metastasis; therefore, we only focused on patients with pT2–4 stage disease (3). In the present study, using a multiple institution patient cohort from a real-world database, we found that for cN0 patients, LND did not improve survival in patients with pT2–4 stage disease.

The assumption of better survival outcomes after performing LND is based on the finding that LN metastasis is a poor risk factor for cancer prognosis in UTUC, which has been well established before (16, 17). Nevertheless, some other studies have failed to find a significantly better prognosis in patients with pN0 and pNx stage UTUC who underwent LND (18, 19). The incidence of LN metastasis increases with higher T stage, with a reported incidence of approximately 60% for ≥pT3 disease (20). It remains difficult to declare that LND is needed if the patient has suspected LN metastasis, not to mention cN0 stage, which reflects a lack of suspicious LN involvement before surgery. Simultaneous LND increases the operation time and increases the risk of perioperative complications, including bleeding and chylous lymphatic leakage, which may be a concern for surgeons (21). In the current study, there was no significant benefit to the survival of patients with UTUC after receiving LND without clinical LN involvement. To ensure cohort homogeneity, we only included patients with ≥pT2 disease, which is considered to have a higher incidence of LN metastasis. We demonstrated that pT stage is a strong prognostic factor for OS, CSS, and DFS outcomes in multivariate analysis, which has been well established in previous studies (22, 23).

The number and extent of LNDs may also be associated with the subsequent prognosis. A meta-analysis showed that the removal of a higher number of LNs was associated with better survival outcomes in patients with UTUC (24). The minimal number of LNs requiring removal is variable, but a previous analysis showed that 8 LNs is the threshold for the improvement of survival in non-metastatic UTUC patients (25). Chappidi et al. demonstrated that removing over 5 LNs can improve CSS compared to that with 1–4 nodes removed (6). In our study, we did not find an association between number of nodes removed and survival, although the mean number of LNs removed (7.46 LNs) was comparable to that in previous studies (OS, p = 0.909; CSS, p = 0.893; and DFS, p = 0.196). Xylinas et al. (20) reported that there will be more missing positive lymph nodes if fewer LNs are removed; therefore, LND is necessary for accurate nodal metastasis status assessment and better postoperative clinical decision-making regarding the follow-up schedule. The distribution of LN metastasis differs between tumor locations; therefore, regional LN template removal according to tumor location is likely more important than the number of LNs that were removed. Kondo et al. also could not find whether the number of LNs removed affected survival in patients with pT2 or higher UTUC; instead, they discovered that the most critical factor regarding whether to remove regional LNs is completely based on the template according to tumor location (26). Matsumoto et al. demonstrated that template-based LND in patients with cN0 UTUC according to tumor anatomical location has better long-term oncological outcomes (27).

**Figure 3:**
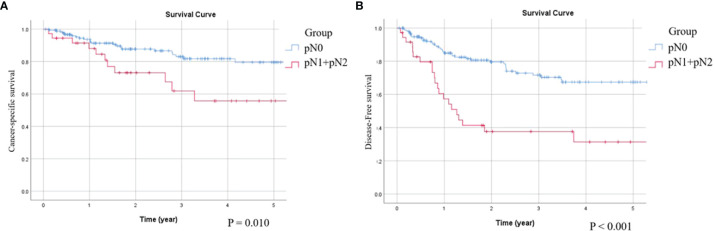
Compare Kaplan-Meier curves in LND (+) group between patients with pN0 and pN1+pN2 by log-rank test. **(A)** Cancer-specific survival, p = 0.010. **(B)** Disease-free Survival, p < 0.001.

The location of UTUC tumors is a prognostic factor that was found to affect survival in previous studies and that ureter tumors have a worse survival rate than tumors in the renal pelvis. One possible hypothesis is that the thicker anatomic barrier of the renal pelvis than that of the ureter leads to more lymphovascular space with a lower chance of spreading out (6, 28). In the present study, we further compared the survival differences between the ureter and renal pelvis tumors in the LND (+) group. No significant differences in OS, CSS, DFS, and BRFS were observed between the renal pelvis and ureter tumors. This demonstrated that performing LND may provide a greater survival benefit in ureter cancer, which is considered to have a higher risk of lymphatic and hematogenous spread than renal pelvis cancer in cN0 stage patients. In the LND (+) group, 18.5% of the patients had pathological LN metastasis. Patients with pathological LN-positive disease (pN1+pN2) had significantly shorter CSS (p = 0.010) and DFS (p < 0.001) than those with no pathological LN metastasis (pN0) (**Figures 3A, B**). The results suggest that meticulous LND during NU may have a therapeutic effect in LN-positive patients, provide accurate staging, and enable postoperative risk stratification for patient counseling. However, we discovered more patients with advanced T stage in the pN (+) subgroup of the LND (+) cohort (T2: 4/47 = 8.5%, T3+T4: 32/147 = 21.8%); the worse survival rate may be due to the more advanced T stage. Pelcovits et al. also observed that some patients with good OS in the pN (+) populations are more likely to have lower stage and lower grade disease (11). The positive predictive value of LND was low at the T2 stage; therefore, for more favorable disease biology (lower stage), NU without LND may be acceptable. The most crucial point is not the number of LNs removed, but the removal of positive LN metastasis. Until now, accurate preoperative clinical staging and assessment of LN invasion in UTUC is difficult (29). Misdiagnosis is a concern for surgeons, and LND may be considered a good method for LN staging, which is a key prognostic factor (24).

Although this study provides important insights into the impact of LND in cN0 stage UTUC, it has some limitations. First, it was performed with a retrospective design including multiple institutions in Taiwan, so a heterogeneous background may exist. Second, this database does not have information regarding the anatomical sites or extent of LND, which may be associated with survival outcomes in UTUC patients. The extent of LND is decided by individual surgeons, so we cannot analyze the effect of the extent of LND on survival outcomes. Furthermore, the lack of centralized image review before surgery may lead to misdiagnosis of clinical N stage. Moreover, because it includes a large cohort from many institutions, even if we have included many covariants, it may have some unavoidable selection bias. Therefore, further prospective studies are warranted to determine the benefits of LND.

## Conclusions

We demonstrated that although pN (+) status has worse survival, performing LND in patients with muscle-invasive cN0 stage UTUC did not show a significant benefit regardless of the number of LNs removed. However, based on the results of the pN (+) subgroup of the LND (+) cohort, it may be reasonable to not perform LND in patients with cT2N0 stage disease due to the low positive predictive value of pN (+). In addition, it may be suggested to perform LND for ureter cancer, which tends to result in lymphatic and hematogenous tumor spreading. Due to the potential increased risk of perioperative complications and considering the accurate staging benefit, a meticulous preoperative plan is needed to decide whether to perform LND.

## Data Availability Statement

The raw data supporting the conclusions of this article will be made available by the authors, without undue reservation.

## Ethics Statement

The studies involving human participants were reviewed and approved by Kaohsiung Medical University Hospital. KMUHIRB-E(I)-20180214. Written informed consent for participation was not required for this study in accordance with the national legislation and the institutional requirements

## Author Contributions

Y-CT and H-YL conceived the project. All authors collected the data. H-YL analyzed the results. H-YL and H-CY drafted the manuscript. H-CY and H-LK edited the manuscript. All authors contributed to the article and approved the submitted version.

## Funding

This study was supported partially by the Ministry of Science and Technology (MOST 109-2314-B-037-095), Kaohsiung Medical University (KMU-KI109002), Kaohsiung Medical University Hospital (KMUH-DK(C)110006), Kaohsiung Medical University Hospital Department of Urology (MOHW110-TDU-B-212-124006), Kaohsiung Medical University Regenerative Medicine and Cell Therapy Research Center (KMU-TC109A02), and Kaohsiung Medical University Center for Liquid Biopsy and Cohort Research (KMU-TC109B05).

## Conflict of Interest

The authors declare that the research was conducted in the absence of any commercial or financial relationships that could be construed as a potential conflict of interest.

## Publisher’s Note

All claims expressed in this article are solely those of the authors and do not necessarily represent those of their affiliated organizations, or those of the publisher, the editors and the reviewers. Any product that may be evaluated in this article, or claim that may be made by its manufacturer, is not guaranteed or endorsed by the publisher.
